# Trends in Mortality in Puerto Rico Between 1979 and 2018: An Analysis of the Puerto Rico Healthcare Reform

**DOI:** 10.1093/geroni/igaa057.347

**Published:** 2020-12-16

**Authors:** Alexis Santos

**Affiliations:** Pennsylvania State University, University Park, Pennsylvania, United States

## Abstract

Between 1993 and 2000, the Government of Puerto Rico decided to transform the role of the government from a provider of healthcare to an insurer. Despite claims about the success of the reform, no study has assessed whether it improved the health of the population or reduced mortality. The aim of this study is to assess whether the implementation of the Puerto Rico Healthcare Reform of 1993 reduced mortality and infant mortality in Puerto Rico in a significant way. I calculated crude death rates (CDR), age-standardized death rates, infant mortality rates, total deaths and life expectancy between 1980 and 2018. I used a quasi-experimental design to study the effect of the implementation of the Puerto Rico Healthcare Reform on these indicators. The primary objective was to estimate changes in trends after 2000. The Age-Specific Mortality Rates have reduced since 1980. The least pronounced change for 2018, in comparison to 1980, was for young adults (20-24 years, 25-29 years, and 30-34 years). The CDR was affected based on the implementation of the reform, but the Infant Mortality Rates was not. The Standardized Death Rate and deaths indicate that there was a small reduction in these indicators. I also found that the gains in life expectancy were concentrated in older adults (aged 65 and older). Analysis of all-cause mortality indicators allows for the evaluation of this healthcare reform. The reduction in mortality in the post-2000 period was not entirely due to the trend that existed before the healthcare reform was implemented.

